# Supplementary material for: gapseq: informed prediction of bacterial metabolic pathways and reconstruction of accurate metabolic models
Source: Genome Biol. 2021 Mar 10;22:81. doi: 10.1186/s13059-021-02295-1 (PMC7949252; doi:10.1186/s13059-021-02295-1)
Supplement: Supplementary file 1 — Additional file 1 Supplementary figures S1-S5. [file 13059_2021_2295_MOESM1_ESM.pdf]

Additional file 1

Supplementary figures S1-5

gapseq: informed prediction of bacterial metabolic pathways and reconstruction of accurate metabolic models

Johannes Zimmermann, Christoph Kaleta, Silvio Waschina

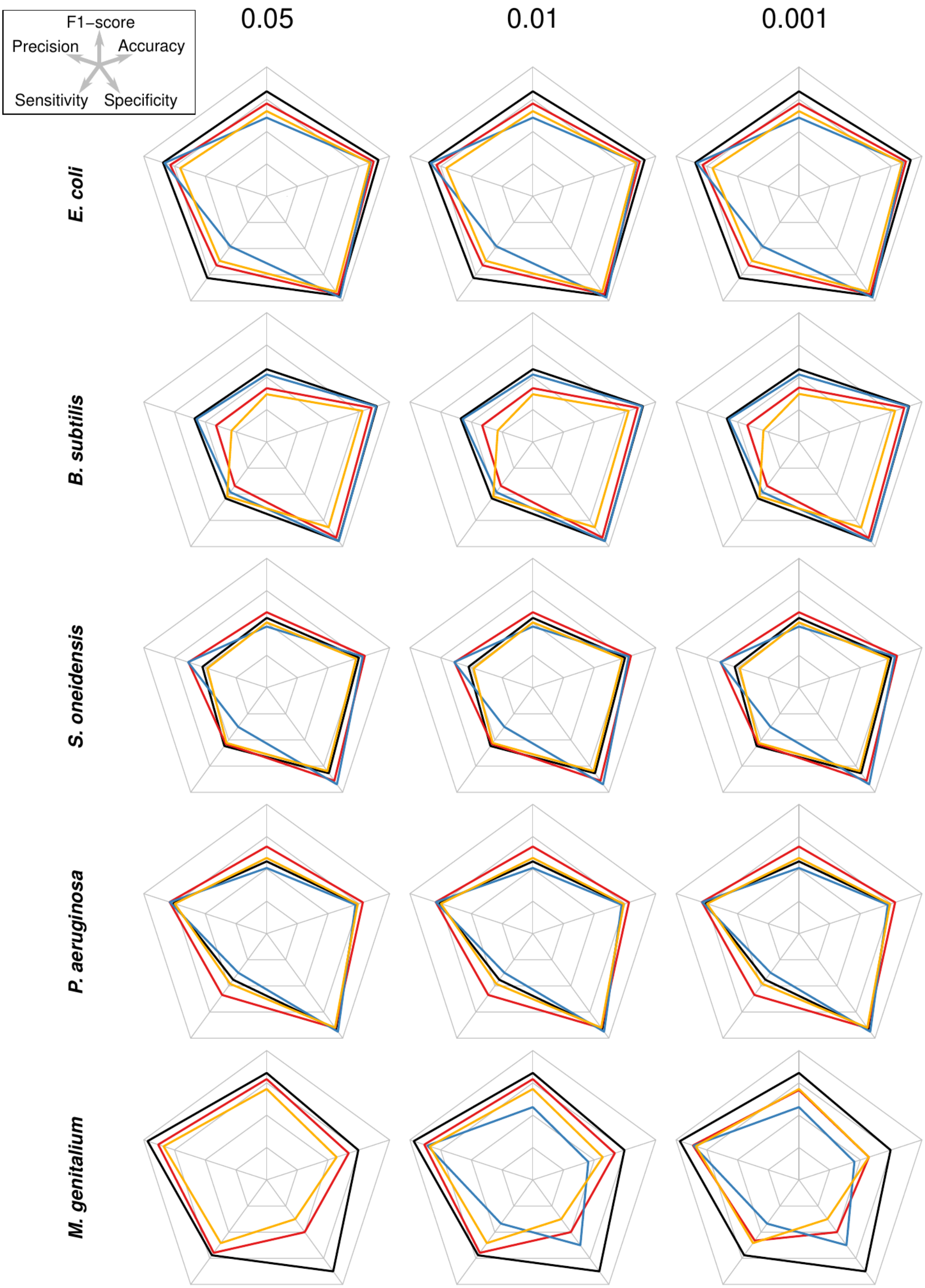

Fig S1 – Results from gene essentiality tests for three different growth rate thresholds.
Genes were predicted to be essential if the predicted growth rate of the respective gene knock-out strain was below 0.05 hr^{-1}, 0.01 hr^{-1} or 0.001 hr^{-1}. Charts in second column (0.01 hr^{-1}) represent the same results as in Figure 2. The radar chart axes display results for five test scores (see top left legend). Axes scales are linear with 0 in the centre and 1 at the corners. The essentiality tests were not feasible with the cutoff 0.05 hr^{-1} for the *M. genitalium* CarveMe reconstruction, as the predicted growth without knockouts was already below this threshold (0.034 hr^{-1}).

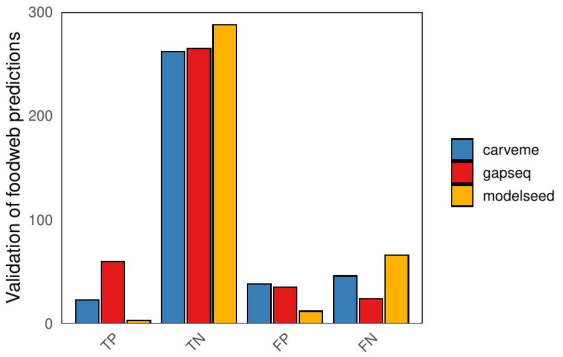

Fig S2 – Validation of substance production and consumption in anaerobic gut communities as referred to Additional file 2: Table S4.

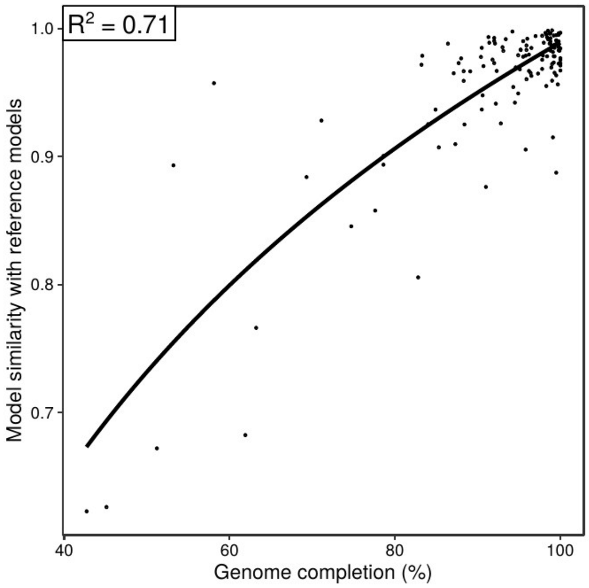

Fig S3 – Similarity of *gapseq* models reconstructed for 127 species-level genome bins (SGBs) from metagenomes compared to models reconstructed for reference genomes (RefSeq Prokaryotic Genomes). The x-axis represents the genome assembly completion of SGBs estimated using the BUSCO software version 4.0.6 (Simao *et al.* (2015, Bioinformatics)). The line shows the result of non-linear regression using a logarithmic function of form y(x)=c+b*log(x). Sequences of SGBs were obtained from Pasolli *et al.* (2019, *Cell*).

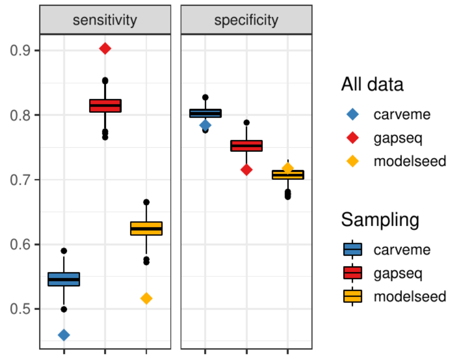

Fig S4 – Re-sampling test of enzyme prediction. Evaluation of enzyme predictions using a re-sampling test (500 iterations), where equal numbers of tests (n=100) for each EC number contributed to the evaluation statistics. Distribution of sensitivity and specificity of CarveMe, ModelSEED, and *gapseq* predictions of sampled data are shown (boxplot) compared with the original data (rhombus).

Fig S5 – Predicted metabolite production and consumption in an anaerobic microbial community using CarveMe, *gapseq*, and ModelSEED model reconstructions. Predictions are based on an agent-based dynamic simulation using the BacArena package within *R* and shown for all 7 time steps of the simulation.

(see next page)

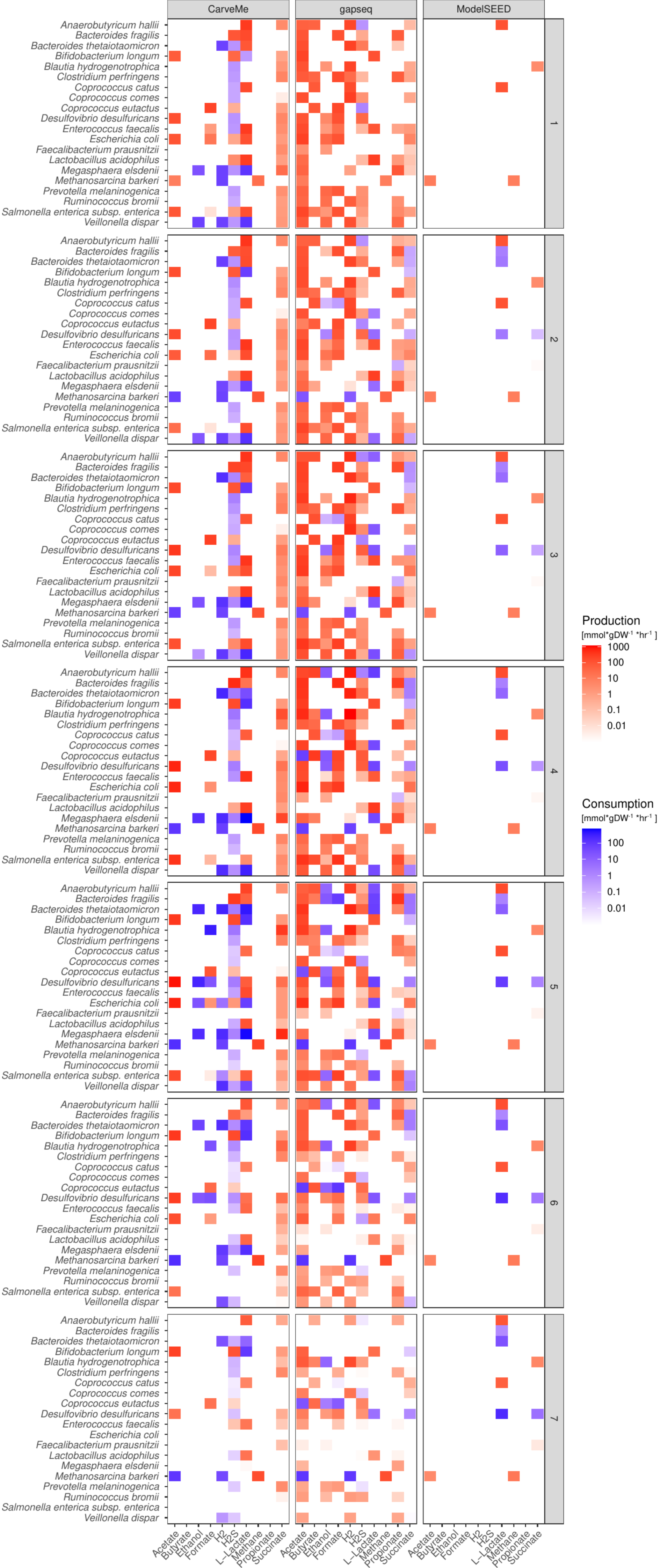
CarveMe
gapseq
ModelSEED
1
2
3
4
5
6
7
Anaerobutyricum hallii
Bacteroides fragilis
Bacteroides thetaiotaomicron
Bifidobacterium longum
Blautia hydrogenotrophica
Clostridium perfringens
Coprococcus catus
Coprococcus comes
Coprococcus eutactus
Desulfovibrio desulfuricans
Enterococcus faecalis
Escherichia coli
Faecalibacterium prausnitzii
Lactobacillus acidophilus
Megasphaera elsdenii
Methanosarcina barkeri
Prevotella melaninogenica
Ruminococcus bromii
Salmonella enterica subsp. enterica
Veillonella dispar
Acetate
Butyrate
Ethanol
Formate
H2
H2S
L-Lactate
Methane
Propionate
Succinate
Production
[mmol*gDW-1 *hr-1]
1000
100
10
1
0.1
0.01
Consumption
[mmol*gDW-1 *hr-1]
100
10
1
0.1
0.01
